# Neonatal withdrawal syndrome following in utero exposure to antidepressants: a disproportionality analysis of VigiBase, the WHO spontaneous reporting database

**DOI:** 10.1017/S0033291722002859

**Published:** 2023-09

**Authors:** C. Gastaldon, E. Arzenton, E. Raschi, O. Spigset, D. Papola, G. Ostuzzi, U. Moretti, G. Trifirò, C. Barbui, G. Schoretsanitis

**Affiliations:** 1 WHO Collaborating Centre for Research and Training in Mental Health and Service Evaluation, Department of Neuroscience, Biomedicine and Movement Sciences, Section of Psychiatry, University of Verona, Verona, Italy; 2 Section of Pharmacology, Department of Diagnostics and Public Health, University of Verona, Verona, Italy; 3 Pharmacology Unit, Department of Medical and Surgical Sciences, University of Bologna, Bologna, Italy; 4 Department of Clinical Pharmacology, St. Olav University Hospital, Trondheim, Norway; 5 Department of Clinical and Molecular Medicine, Norwegian University of Science and Technology, Trondheim, Norway; 6 Department of Psychiatry, The Zucker Hillside Hospital, Northwell Health, Glen Oaks, NY, USA; 7 Department of Psychiatry, Zucker School of Medicine at Northwell/Hofstra, Hempstead, NY, USA; 8 Department of Psychiatry, Psychotherapy and Psychosomatics, Hospital of Psychiatry, University of Zurich, Zurich, Switzerland

**Keywords:** Abstinence syndrome, antidepressants, neonates, poor neonatal adaptation syndrome, pregnancy, withdrawal syndrome

## Abstract

**Background:**

Evidence on neonatal withdrawal syndrome following antidepressant intrauterine exposure is limited, particularly for antidepressants other than selective serotonin reuptake inhibitor (SSRIs).

**Methods:**

In our case/non-case pharmacovigilance study, based on VigiBase^®^, the WHO database of suspected adverse drug reactions, we estimated reporting odds ratio (ROR) and the Bayesian information component (IC) with 95% confidence/credibility intervals (CI) as measures of disproportionate reporting of antidepressant-related neonatal withdrawal syndrome. Antidepressants were first compared to all other medications, then to methadone, and finally within each class of antidepressants: SSRIs, tricyclics (TCA) and other antidepressants. Antidepressants were ranked in terms of clinical priority, based on semiquantitative score ratings. Serious *v.* non-serious reports were compared.

**Results:**

A total of 406 reports of neonatal withdrawal syndrome in 379 neonates related to 15 antidepressants were included. Disproportionate reporting was detected for antidepressants as a group as compared to all other drugs (ROR: 6.18, 95% CI 5.45–7.01, IC: 2.07, 95% CI 1.92–2.21). Signals were found for TCAs (10.55, 95% CI 8.02–13.88), followed by other antidepressants (ROR: 5.90, 95% CI 4.74–7.36) and SSRIs (ROR: 4.68, 95% CI 4.04–5.42). Significant disproportionality emerged for all individual antidepressants except for bupropion, whereas no disproportionality for any antidepressant was detected *v.* methadone. Eleven antidepressants had a moderate clinical priority score and four had a weak one. Most frequent symptoms included respiratory symptoms (*n* = 106), irritability/agitation (*n* = 75), tremor (*n* = 52) and feeding problems (*n* = 40).

**Conclusions:**

Most antidepressants are associated with moderate signals of disproportionate reporting for neonatal withdrawal syndrome, which should be considered when prescribing an antidepressant during pregnancy, irrespective of class.

## Introduction

Withdrawal syndrome in neonates following in utero exposure to antidepressants was first reported several decades ago (Kent & Laidlaw, 1995; Spencer, 1993). More comprehensive assessments of these clinical manifestations have later become available (Levinson-Castiel, Merlob, Linder, Sirota, & Klinger, 2006), in line with increasing prescription trends of antidepressants in women during pregnancy (Sun et al., 2019). Antidepressant-related discontinuation symptoms in neonates were first described as the neonatal abstinence syndrome (Levinson-Castiel et al., 2006), whereas more recently literature adopted the term withdrawal symptoms (Wang & Cosci, 2021). Currently, there is no unanimous consensus regarding terminology. There is also a lack of consistent definition of the syndrome and what symptoms are involved. Although the term neonatal adaptation syndrome might be more correct from a mechanistic point of view, we have chosen to use the term neonatal withdrawal syndrome in this article, as this term (and variants of it) is the predominating term used in the database from which we have retrieved data (for details, see Methods section). Antidepressant-related withdrawal syndrome in neonates has mostly received attention after maternal treatment with selective serotonin reuptake inhibitors (SSRIs) and venlafaxine (Shea et al., 2021; Wang & Cosci, 2021).

A previous pharmacovigilance study assessed reporting patterns for withdrawal syndrome in neonates of mothers prescribed SSRIs recorded in VigiBase^®^, the World Health Organization (WHO) global individual case safety reports (ICSRs) database up to 2003 (Sanz, De-las-Cuevas, Kiuru, Bate, & Edwards, 2005). In a total of 102 ICSRs of SSRI-associated neonatal withdrawal syndrome, four SSRIs were recorded: paroxetine, fluoxetine, sertraline and citalopram. The Bayesian estimate of the information component (IC), showed a significantly disproportionate reporting, i.e. potential association, of neonatal withdrawal effects for all four SSRIs compared to other medications in the database (Sanz et al., 2005). Additionally, findings implied higher disproportionality for paroxetine as compared to the other three SSRIs, possibly related to its high affinity for the muscarinic receptors, which may increase the risk of cholinergic withdrawal syndrome, and its short half-life may increase the risk of any withdrawal symptom (Sanz et al., 2005). According to observational studies and meta-analyses of observational studies, approximately one third of the neonates of mothers treated with SSRIs/venlafaxine during pregnancy develop a withdrawal syndrome (Shea et al., 2021; Wang & Cosci, 2021). Very common reported symptoms include respiratory (e.g. respiratory depression, apnea or dyspnea), neuromuscular and central nervous system (e.g. convulsions, agitation, somnolence or hypertonia/hypotonia), cardiovascular (e.g. circulatory failure or atrial septal defect) and gastrointestinal symptoms (e.g. vomiting, feeding disorders or diarrhoea) that may last up to two weeks postpartum (Wisner et al., 2009).

Although the potential of antidepressants to cause withdrawal syndrome in neonates is thoroughly described for SSRIs and venlafaxine, data on other antidepressants remain limited. Moreover, evidence from comparisons of the risk of neonatal withdrawal syndrome between antidepressants is barely available, mainly due to the limited size of the available cohorts (Wang & Cosci, 2021).

Pharmacovigilance databases and pharmacovigilance studies comprise a cornerstone data source to assess adverse drug reactions (ADRs) in real-world settings (Moore, Morrow, Dormuth, & Mintzes, 2020; Nakamura, 2016; van Puijenbroek et al., 2002). In fact, the analysis of large pharmacovigilance with disproportionality analysis (Bate, Lindquist, & Edwards, 2008), allows detecting clinically important safety signals for somatic as well as psychotropic medications (Poluzzi, Raschi, Moretti, & De Ponti, 2009; Raschi et al., 2013; Rees, Chyou, & Nishtala, 2020; Vickers-Smith et al., 2020), and complemented the evidence gathered from pre-marketing clinical trials and observational studies (de Leon, Ruan, Verdoux, & Wang, 2020; Gastaldon, Raschi, Kane, Barbui, & Schoretsanitis, 2021; Trenque et al., 2013; Umetsu et al., 2015). Given the inherent challenges of pregnancy and neonatal research such as the ethical justification for inclusion in randomized trials (Kaye, 2019), available approaches to assess antidepressant-related withdrawal syndrome in neonates other than pharmacovigilance data and analyses of medical birth registers are limited.

The aim of this study was to explore the potential association between maternal antidepressant treatment and withdrawal syndrome in neonates, and to investigate the comparative reporting between subgroups of antidepressants as well as between individual agents.

## Methods

The protocol for this study was published in advance on OpenScienceFramework (https://osf.io/kjwmr/). We conducted a disproportionality analysis through the case/non-case study design (Faillie, 2019). ICSRs from VigiBase^®^, the largest worldwide pharmacovigilance database containing over 28 million ICSRs on suspected ADRs from 140 member countries, were analyzed. Further details on the items contained in ICSRs are provided on the Uppsala Monitoring Centre (UMC) website (Uppsala Monitoring Centre, 2017). All deduplicated ICSRs recorded in VigiBase^®^ from inception to 31 August 2021 were included. Cases were reports of withdrawal symptoms in neonates, i.e. individuals of 1–27 days of age identified using the preferred terms (PT) ‘withdrawal syndrome’, ‘drug withdrawal syndrome’, ‘drug withdrawal syndrome neonatal’, ‘antidepressants discontinuation syndrome’, ‘drug withdrawal headache’, ‘drug withdrawal convulsions’, ‘drug withdrawal maintenance therapy’, and the sub-Standardized MedDRA Queries (sub-SMQs) ‘drug withdrawal’. These PTs and sub-SMQs were considered as they are the most representative of withdrawal syndrome, although there is no consensus in the literature regarding terminology and also a lack of consistent definition of the syndrome and what symptoms are involved. In fact, poor neonatal adaptation syndrome is also very usual in literature (Corti et al., 2019; Hendson, Shah, & Trkulja, 2021; Kautzky, Slamanig, Unger, & Hoflich, 2022; Rommel et al., 2022), although there is not a PT for it. Non-cases were all reports of ADRs other than withdrawal symptoms in neonates. Removal of duplicates was performed before analyses based on standardized VigiBase-inherent algorithms. We searched for reports indicating any of the following 27 antidepressants as suspected or interacting drug, following the Anatomical Therapeutic Classification (ATC) system (WHO, 2021). Tricyclics (TCAs) were amitriptyline, clomipramine, desipramine, doxepin, imipramine, lofepramine and nortriptyline; SSRIs were citalopram, escitalopram, fluoxetine, fluvoxamine, paroxetine and sertraline; and ‘other’ antidepressants were agomelatine, bupropion, desvenlafaxine, duloxetine, mianserin, milnacipran, mirtazapine, nefazodone, reboxetine, Saint John's wort (*Hypericum perforatum*), trazodone, venlafaxine, vilazodone and vortioxetine. These antidepressants are among the most commonly prescribed antidepressants worldwide based on current pharmacoepidemiological data (Cebron Lipovec, Anderlic, & Locatelli, 2022; Lalji, McGrogan, & Bailey, 2021; medicaid.gov, 2022).

Our primary outcome included two types of disproportionality analyses: the reporting odds ratio (ROR) (van Puijenbroek et al., 2002) and the Bayesian IC (Bate et al., 1998), together with 95% confidence (for ROR) and credibility (for IC) intervals (95% CIs), for antidepressants with more than three reports of neonatal withdrawal syndrome. Established thresholds for significance were adopted (i.e. lower limit of the 95% CI >1 and >0 for ROR and IC, respectively) and we considered a potential safety signal when both ROR and IC were statistically significant, to increase the robustness of the results. As main disproportionality analysis, we estimated the ROR and IC of neonatal withdrawal syndrome for antidepressants as a whole, for each antidepressants class (TCAs, SSRIs, and others), and for each individual antidepressant compared to all other drugs (except antidepressants) registered in the VigiBase^®^. We, then, performed secondary analyses using methadone, an established positive control, as comparator. The selection of the positive control aims for a medication that is well-known to produce symptoms similar to those investigated in the study hypothesis, although we fully acknowledge that the neonatal withdrawal syndrome for antidepressants clinically differs from the neonatal abstinence syndrome related to opioids (Bakhireva et al., 2022). Methadone is the first-line opioid-substitution treatment in pregnant women and it is known to induce neonatal withdrawal syndrome (Devlin et al., 2021); thus, clinicians may actually be more primed to report neonatal withdrawal syndrome for methadone. We also assessed the intraclass disproportionality of withdrawal syndrome by comparing individual antidepressants with all other antidepressants of the same class. As such, ROR and IC for all antidepressants as a whole, for each antidepressants class (TCAs, SSRIs, or others), and for each individual antidepressant were estimated. The rationale of this approach was to explore differences within the same antidepressant class and potential drivers of the detected signals. This type of approach has been previously used in pharmacovigilance, also in the field of psychopharmacology (Cepaityte, Siafis, & Papazisis, 2021; Zhou et al., 2022).

Antidepressants with disproportionate reporting for neonatal withdrawal syndrome were classified in terms of clinical prioritization based on a semiquantitative score assessing four features: (1) number of withdrawal syndrome reports out of the total number of ADR reports (>10%: 2 points, 5–10%: 1 point, 0–4%: 0 points); (2) number of withdrawal syndrome reports with antidepressant after removing reports indicating potential confounders (i.e. co-medications possibly related to withdrawal syndrome) out of the total cases of withdrawal syndrome (⩽71%: 2 points, 51–70%: 1 point, ⩽50%: 0 points); (3) the consistency of statistically significance of ROR and IC (if ROR and IC were statistically significant in the main, in the intraclass analysis and in the analysis with methadone as comparator: 2 points; in two analyses: 1 point, in only one analysis: 0 points); (4) magnitude of the lower 95% CI of ROR (>10: 1 point, 0–10: 0 points). Subsequently, antidepressants with scores ranging 0–1, 2–5 or 6–7 were classified as of weak (green light), moderate (yellow light) or strong (red light) clinical priority, respectively. Details and thresholds for each criterion and rating system are described in the online Supplementary Table S1.

Comparisons between neonates with serious *v.* non-serious withdrawal syndrome with regard to sex, age, symptom duration, maternal antidepressant daily dose, duration of maternal antidepressant treatment and maternal co-medications were conducted using non-parametrical tests. According to the WHO definitions, serious reactions refer to ADR resulting in death, requiring hospital admission or prolongation of existing hospital stay, life-threatening event, congenital anomalies or with permanent sequelae (Edwards & Aronson, 2000). Cases of missing data were not included in the comparisons. We registered the most common reactions co-reported with withdrawal syndrome to characterize the core symptoms of neonatal antidepressant-related withdrawal syndrome. Further, we performed comparisons between neonates with serious *v.* non-serious withdrawal syndrome with regard to sex, age, symptom duration, maternal antidepressant daily dose, duration of maternal antidepressant treatment and maternal co-medications in the subgroup of neonates of mothers exclusively treated with antidepressants. Networks of co-occurring symptoms in a subset of reports without potential confounders were visualized using Gephi, version 0.9.2 for Windows (https://gephi.org/).

## Results

A total of 406 reports of antidepressant-related withdrawal syndrome referring to 379 neonates (139 females, 36.7%) were identified. Demographic and clinical characteristics are provided in Table 1. The median duration of maternal antidepressant treatment was 36.7 (interquartile range 28.0–39.0) weeks; the median reported duration of the withdrawal symptoms was 7.0 (interquartile range 3.0–11.0) days, with 318 reports (83.9%) being classified as serious.

**Table 1.** Characteristics of the cases

Characteristics of the cases	*n* = 379
Neonatal sex: females, *n* (%)	139 (36.7%)
Neonatal age (days), median (Q1–Q3)	1.0 (0.0–2.0)
Country	
Europe	261 (68.9%)
North America	75 (19.8%)
Asia	33 (8.7%)
Oceania	9 (2.4%)
Other	1 (0.3%)
Maternal dose (DDD), median (Q1–Q3)	1.00 (0.7–2.0)
Maternal duration of treatment (weeks), median (Q1–Q3)	36.71 (28.0–39.0)
Duration of the neonatal withdrawal symptoms (days), median (Q1–Q3)	7.0 (3.0–11.0)
Serious reaction (*n*, %)^a^	318 (83.9%)
With at least one reported psychotropic maternal co-medication, *n* (%)	209 (55.1%)
With mood stabilizers (*n*)	36 (9.5%)
With benzodiazepines (*n*)	114 (30.1%)
With other antidepressants (*n*)	45 (11.9%)
With antipsychotics (*n*)	88 (23.2%)
With opioids (*n*)	51 (13.5)

DDD, defined daily dose; n, number of neonates; Q1, first quartile; Q3, third quartile.

a Data on symptom severity were available for 330 patients.

We found at least four reports of withdrawal syndrome only for 15 antidepressants (Table 2). The most frequently reported suspected antidepressants there were paroxetine (*n* = 70), venlafaxine (*n* = 67), sertraline (*n* = 52), fluoxetine (*n* = 50), clomipramine (*n* = 47), escitalopram (*n* = 34), citalopram (*n* = 33), amitriptyline (*n* = 14) and mirtazapine (*n* = 10). The median overall defined daily dose (DDD) was 1.00 (0.75–2.00) mg/day. The reported doses for each antidepressant are shown in the online Supplementary Table S2. Approximately half of the mothers (55.2%) used at least one psychotropic drug concomitantly; 30.1% of the mothers were co-prescribed benzodiazepines, 23.2% antipsychotics, 13.5% opioids, 11.9% more than one antidepressant and 9.5% mood stabilizers.

**Table 2.** Reporting odds ratios (ROR) and information components (IC) for antidepressant-related neonatal withdrawal syndrome by class of antidepressant and for each antidepressant

Drug	*n* cases	*n* non-cases	ROR	Lower 95% CI	Upper 95% CI	IC	Lower 95% CI	Upper 95% CI
Antidepressants (All)	379	2817	6.18	5.45	7.01	2.07	1.92	2.21
Tricyclic antidepressants	69	238	10.55	8.02	13.88	2.93	2.57	3.25
SSRIs	238	2060	4.68	4.04	5.42	1.88	1.69	2.05
Other antidepressants	99	620	5.91	4.74	7.36	2.27	2.00	2.54
Tricyclic antidepressants								
Amitriptyline	14	100	4.89	2.79	8.58	1.97	1.12	2.63
Clomipramine	47	117	14.40	10.22	20.30	3.22	2.78	3.60
Doxepin	5	10	17.38	5.93	50.92	2.58	1.05	3.57
SSRIs								
Citalopram	33	307	3.79	2.64	5.46	1.74	1.20	2.19
Escitalopram	34	293	4.10	2.86	5.87	1.83	1.31	2.28
Fluoxetine	50	460	3.88	2.88	5.22	1.77	1.34	2.14
Fluvoxamine	7	33	7.38	3.26	16.72	2.21	0.95	3.08
Paroxetine	71	641	4.00	3.11	5.15	1.80	1.45	2.12
Sertraline	51	378	4.82	3.58	6.50	2.04	1.61	2.41
Other antidepressants								
Bupropion	6	96	2.17	0.95	4.96	0.95	−0.42	1.88
Duloxetine	8	84	3.31	1.60	6.86	1.46	0.29	2.29
Mianserin	4	5	27.79	7.45	103.61	2.58	0.84	3.66
Mirtazapine	10	62	5.62	2.88	10.99	2.06	1.03	2.81
Trazodone	6	25	8.34	3.42	20.38	2.25	0.87	3.17
Venlafaxine	67	338	7.19	5.50	9.40	2.51	2.14	2.83

CI, confidence/credibility interval; IC, information component; ROR, reporting odds ratio; SSRI, selective serotonin reuptake inhibitors.

**Fig. 1.**
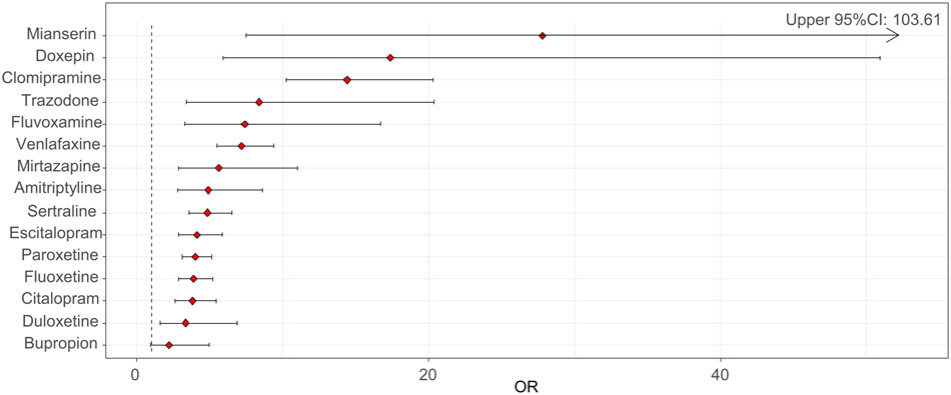
Reporting odds ratios (RORs) and 95% confidence intervals (CI) for each antidepressant (ROR > 1 indicates an increased withdrawal reporting associated with antidepressants) – all other drugs were considered as a comparator.

We found a statistically significant reporting disproportionality for antidepressants as a whole, with a ROR of 6.18 (95% CI 5.45–7.01) and an IC of 2.07 (95% CI 1.92–2.21). Across AD classes, strongest RORs were found for TCAs (10.55, 95% CI 8.02–13.88; IC: 2.93, 95% CI 2.57–3.25), followed by other antidepressants (ROR: 5.90, 95% CI 4.74–7.36; IC: 2.27, 95% CI 1.97–2.54) and SSRIs (ROR: 4.68, 95% CI 4.04–5.42; IC: 1.88, 95% CI 1.69–2.05). We detected a significant disproportionate reporting for all investigated antidepressants except for bupropion (Table 2, Fig. 1), with the highest RORs for mianserin, doxepin, clomipramine and trazodone (Fig. 1, Table 2).

When using methadone as the active comparator, the safety signal for antidepressants overall was not significant (ROR: 0.07, 95% CI 0.06–0.10). Likewise, when analyzing by class no disproportionalities *v.* methadone were reported for TCAs (ROR: 0.16, 95% CI 0.11–0.23), for other antidepressants (ROR: 0.09, 95% CI 0.06–0.12) or for SSRIs (ROR: 0.06, 95% CI 0.05–0.08). When analyzing each antidepressant separately compared to methadone, no significant disproportionate reporting was detected (online Supplementary Table S3).

In the intraclass analyses, we did not detect any signals for any antidepressant compared to other antidepressants from the same class (online Supplementary Tables S4–S6).

The comparison of neonates with serious *v.* non-serious withdrawal syndrome (318 *v.* 12 neonates, respectively) did not reveal any significant differences, but there was a trend for longer duration of antidepressant maternal treatment in neonates with serious reactions (*p* = 0.08, Table 3). No differences were reported in the comparison of neonates with serious *v.* non-serious withdrawal syndrome in the subgroup of neonates of mothers only prescribed antidepressants without other psychotropic co-medications (*n* = 158, online Supplementary Table S7).

**Table 3.** Comparison between serious and non-serious reactions

	Serious reactions	Non-serious reactions	OR (95% CI)	*p* value
*N*	318	12		
Neonatal sex: Females, *n* (%)	115 (36.2)^a^	4 (33.3)^b^	1.00 (0.20–4.31)	1.00
Neonatal age (days), median (Q1–Q3)	1.0 (0.0–1.0)	1.0 (0.0–1.3)	NA	0.93
Maternal DDD, median (Q1–Q3)	1.0 (1.0–2.0)^c^	1.0 (0.4–1.8)^d^	NA	0.49
Duration of the maternal antidepressant treatment (days), median (Q1–Q3)	258.0 (222.0–271.8)^e^	85.0 (83.5–86.5)^f^	NA	0.08
Duration of neonatal withdrawal syndrome (days), median (Q1–Q3)^d^	7.0 (3.0 11.0)^g^	2.5 (1.0–5.5)^h^	NA	0.18
With mood stabilizers (*n*)	32	1	1.23 (0.17–54.61)	1.00
With benzodiazepines (*n*)	99	2	2.26 (0.47–21.55)	0.36
With other antidepressants (*n*)	41	1	1.63 (0.23–71.75)	1.00
With antipsychotics (*n*)	78	2	1.62 (0.33–15.55)	0.74
With opioids (*n*)	43	1	1.72 (0.24–75.71)	1.00

CI, confidence interval; DDD, defined daily dose; NA, not applicable; OR, odds ratio; Q1, first quartile; Q3, third quartile.

a Missing data for 31 neonates.

b Missing data for two neonates.

c Data available for 180 neonates.

d Data available for 7 neonates.

e Data available for 86 neonates.

f Data available for two neonates.

g Data available for 62 neonates.

h Data available for four neonates.

Among the 15 antidepressants for which reports were available, 11 were classified as having a moderate clinical priority score for neonatal withdrawal syndrome (amitriptyline, clomipramine, doxepin, escitalopram, paroxetine, sertraline, fluvoxamine, mianserin, mirtazapine, trazodone and venlafaxine), whereas four were classified as having a weak priority (bupropion, citalopram, duloxetine, fluoxetine). None were classified as having a strong clinical priority for neonatal withdrawal syndrome (online Supplementary Table S8).

**Fig. 2.**
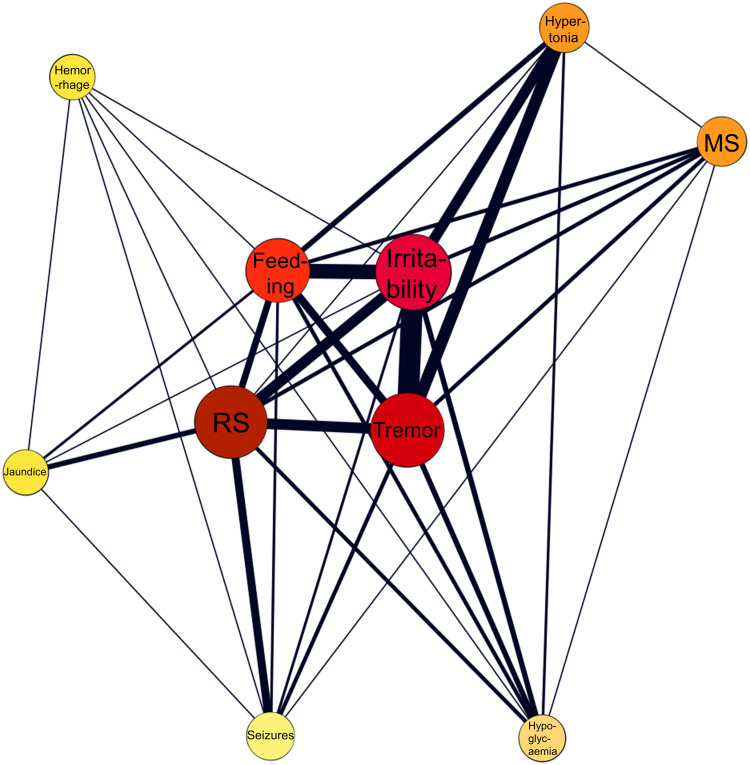
Network analysis of co-occurring symptoms in a subset of 69 neonates with antidepressant monotherapy and without confounding psychotropic medications. The color and the size of the nodes are proportionate to the frequency of the symptoms, whereas the thickness of the edges is proportionate to the frequency of co-occurrences. Feeding, feeding problems; MS, muscle symptoms; RS, respiratory symptoms.

Most frequently reported symptoms included respiratory symptoms (*n* = 106, 26.11%), irritability/agitation (*n* = 75, 18.47%), tremor (*n* = 52, 12.81%), feeding problems (*n* = 40, 9.85%) and seizures (*n* = 33, 8 13%). The network of co-occurring symptoms in a subset of 69 neonates without psychotropic co-medications and at least two co-reported symptoms is provided in Fig. 2.

## Discussion

Our worldwide pharmacovigilance study provides real-world data on neonatal withdrawal syndrome suspectedly reported with antidepressants. For the majority of these, this is the first time that evidence implying a potential for neonatal withdrawal syndrome comes into light. Overall, neonatal withdrawal syndrome was disproportionately reported for all antidepressants except bupropion compared to all other (non-antidepressant) drugs in the database, albeit with some notable differences.

Among antidepressant classes, neonatal withdrawal syndrome was more frequently reported for TCAs than for SSRIs or other antidepressants. This finding deserves special attention as, to date, knowledge for TCA-related neonatal syndrome mainly derives from limited case series (ter Horst et al., 2012). In fact, contrasting the availability of reports for SSRI-related neonatal withdrawal syndrome (Wang & Cosci, 2021), there is a lack of literature on the risk of neonatal withdrawal syndrome for most TCAs. Our study provides evidence on the risk of neonatal withdrawal syndrome for clomipramine, amitriptyline and doxepin, which were ranked with moderate clinical priority based on our assessment. Although there are previous reports of withdrawal syndrome for the most serotoninergic TCA, clomipramine (Bloem, Lammers, Roofthooft, De Beaufort, & Brouwer, 1999; Singh, Gulati, Narang, & Bhakoo, 1990; ter Horst et al., 2012), we are not aware of any published reports for amitriptyline or doxepin. We also provide first-time evidence for several other (non-TCA/non-SSRI) antidepressants and their role in the neonatal withdrawal syndrome. This is particularly important because as for TCAs, the available literature is generally lacking. In our main analysis for other antidepressants, there was a disproportionate reporting of neonatal withdrawal syndrome compared to non-antidepressant drugs, with the highest absolute number of cases reported for venlafaxine. Specifically, our findings suggest disproportionate reporting of neonatal withdrawal syndrome for duloxetine, mianserin, mirtazapine, trazodone and venlafaxine. Except for duloxetine, the rest of these medications were ranked with a moderate clinical priority for neonatal withdrawal syndrome. Although there is no formal consensus on the approaches for signal prioritization, especially in terms of thresholds, these criteria have been broadly adopted in pharmacovigilance to highlight signals of clinical interest, namely those with robust disproportionality across analyses (Gatti, Antonazzo, Diemberger, De Ponti, & Raschi, 2021; Raschi, Fusaroli, Ardizzoni, Poluzzi, & De Ponti, 2021; Raschi et al., 2013; Salvo et al., 2014). It is important to clinically prioritize disproportionality signals to provide further clarification assisting clinicians in the prescription or monitoring of antidepressant treatment in pregnant women. Venlafaxine has previously been associated with the neonatal withdrawal syndrome (Holland & Brown, 2017; Wang & Cosci, 2021), but our evidence suggests, for the first time, that mianserin, mirtazapine and trazodone may also be involved.

When assessing the differential reporting between individual antidepressants for neonatal withdrawal syndrome, data on the ability of antidepressants to enter fetal and new-born circulation via umbilical cord or breast milk could be useful to interpret patterns (Schoretsanitis et al., 2021). For example, the strong disproportionality for mianserin or venlafaxine may be explained by a high penetration rate into amniotic fluid (Schoretsanitis et al., 2021).

Interestingly, there was a sex imbalance in our cohort with more reports of antidepressant-related neonatal withdrawal syndrome in males than females. The fetal male sex has been previously associated with higher risk of maternal peripartum depressive symptoms compared to the female sex (Cowell, Colicino, Askowitz, Nentin, & Wright, 2021). Potential mechanisms underlying this association may include the involvement of hormonal circuits, e.g. lower pregnancy estrogen levels of women carrying males (Toriola et al., 2011). Additionally, the fetal male sex has been previously associated with higher risk of obstetric complications (Mortensen et al., 2020). Put together, obstetric complications and antidepressant-related neonatal withdrawal syndrome may be related to the well-established notion of poorer adaptation of male than female fetuses to suboptimal uterine environments (DiPietro & Voegtline, 2017).

The comparison between neonates with reports of serious *v.* non-serious antidepressant-related neonatal withdrawal syndrome did not reveal any difference. There was a trend for the possible duration of antidepressant treatment; serious reactions had a mean maternal treatment duration of more than 36 weeks, compared to 12 weeks in non-serious reactions, suggesting that new-borns with serious reactions might have been exposed to antidepressants for almost the whole duration of the pregnancy. The lack of data regarding the timepoint of initiating antidepressant treatment during pregnancy (for treatment duration shorter than average pregnancy duration) hampers the better understanding of this finding.

Regarding clinical manifestations of antidepressant-related neonatal syndrome, the main symptoms included respiratory symptoms, irritability/agitation, tremor, feeding problems and seizures. However, seizures were not included in the core symptoms in women on antidepressant monotherapy. Thus, the rather high proportion of seizures in our main analysis (13%) may in fact be due to polypharmacy. Specifically, in our sample approximately every other neonate had been exposed to more than one psychotropic medication. Previous literature has highlighted the role of drug-drug interactions leading to more severe neonatal withdrawal symptoms (Bakhireva et al., 2022); thus, we performed a network analysis in a subset of neonates with antidepressant monotherapy and at least two co-reported symptoms. The aim of this network analysis was to provide a more representative cluster of withdrawal syndrome symptoms in neonates exposed to antidepressant monotherapy. As in the total sample, the network analysis in the subset of neonates exposed to antidepressant monotherapy suggested a quartet of core symptoms including respiratory symptoms, irritability/agitation, tremor and feeding problems. There is a strong overlap between this symptom cluster and the clinical characterisation provided for SSRI-related neonatal withdrawal syndrome previously (Wang & Cosci, 2021).

Our findings need to be considered in the light of well-known limitations, mainly inherent to pharmacovigilance research, e.g. the inability to make firm causality, the various and unknown degrees of underreporting between individual drugs, the lack of more specific clinical information and of the prescription patterns of antidepressants during pregnancy worldwide that would have allowed for estimation of absolute incidence rates (Aagaard & Hansen, 2009; Hazell & Shakir, 2006; Shalviri, Mohammad, Majdzadeh, & Gholami, 2007; van der Heijden, van Puijenbroek, van Buuren, & van der Hofstede, 2002). Further, the reporting of adverse reactions usually increases following safety alerts or media attention, resulting in a potential notoriety bias (Pariente, Gregoire, Fourrier-Reglat, Haramburu, & Moore, 2007), which may be also related to stigma toward psychotropic medications (Davis, McDaniel, Wang, & Garza, 2022). However, this is rather unlikely in our analyses, where antidepressants with the strongest disproportionality were not the ones with the highest absolute number of reports. In fact, it could be argued that methadone may be more affected by notoriety bias and thus over-reported. Moreover, the comparison with methadone needs to be interpreted with caution as there are distinct clinical differences between the neonatal withdrawal syndrome for antidepressants and the neonatal opioid withdrawal syndrome (Bakhireva et al., 2022). Despite limitations, pharmacovigilance research represents an essential method to monitor drug safety (Khouri et al., 2021), especially when clinical trials and observational studies are lacking, such as in this field. Additionally, a major challenge derives from the lack of a consensus regarding terminology for neonatal withdrawal syndrome. We used available PTs to identify cases of neonatal withdrawal syndrome, for which other terms such as poor neonatal adaptation syndrome are also very common (Corti et al., 2019; Hendson et al., 2021; Kautzky et al., 2022; Rommel et al., 2022) and a consistent definition is lacking. Besides, the availability of additional demographic and clinical information would have allowed us to better characterize the study sample. For example, although data on maternal antidepressant treatment duration was available, it was not clear when antidepressant treatment was started during pregnancy. Moreover, as the VigiBase exclusively contains spontaneous reports of ADRs and we did not have access to data of total antidepressant exposure in pregnant women during the study period, we were not able to provide estimates of incidence for antidepressant-related neonatal withdrawal syndrome. Furthermore, future research may explore the potential of less commonly or off-label prescribed antidepressants. Last, the subsample of neonates with non-serious reports of antidepressant-related withdrawal syndrome was small and thus the comparison of serious *v.* non-serious reports may well have been underpowered, with an inherent risk of not revealing differences as statistically significant.

Although we acknowledge that our findings are at the exploratory phase and replication is required, we consider that our findings are of importance both for clinicians treating pregnant women and for neonatologists. When balancing potential benefits and risks for the prescription of antidepressants during pregnancy clinicians should not underestimate the risk of neonatal withdrawal syndrome with any type of antidepressant. In fact, here we provide evidence that, in addition to the described concerns for SSRI effects on neonates, the risk of neonatal withdrawal syndrome may be substantial also for non-SSRI antidepressants. On the other hand, while evidence on disproportionate reporting of antidepressant-related neonatal withdrawal syndrome is important, it must be weighed against the importance of adequate treatment of perinatal depression which may also involve antidepressants; untreated perinatal depression may have dramatic effects on maternal and fetal health (Jahan et al., 2021). We also highlighted that the reporting of withdrawal syndrome may increase in neonates exposed to the antidepressants for longer periods during pregnancy; therefore, during long-term treatment during pregnancy clinicians should acknowledge the risk of neonatal withdrawal syndrome, informing mothers about this possibility. Moreover, guidelines on the pharmacological treatment of depressive episodes during pregnancy (Molenaar, Kamperman, Boyce, & Bergink, 2018) should integrate information on the antidepressant-related neonatal withdrawal syndrome. Consequently, within the risk/benefit assessment of antidepressant treatment in pregnant women, knowledge on the antidepressant-related neonatal withdrawal syndrome risk should be taken into account.

Finally, we hope that this work will orientate future research activity, ultimately improving the understanding of antidepressant-related neonatal withdrawal syndrome, including its clinical features as well as its pharmacodynamic and pharmacokinetic correlates (ter Horst et al., 2012).

## Supplementary material

For supplementary material accompanying this paper visit https://doi.org/10.1017/S0033291722002859. click here to view supplementary material

## UMC statement

VigiBase, the WHO global database of ICSRs is the source of the information; the information comes from a variety of sources, and the probability that the suspected adverse effect is drug-related is not the same in all cases; the information does not represent the opinion of the UMC or the WHO.
