# Supplementary material for: Neonatal withdrawal syndrome following in utero exposure to antidepressants: a disproportionality analysis of VigiBase, the WHO spontaneous reporting database
Source: Psychol Med. 2022 Sep 21;53(12):5645–53. doi: 10.1017/S0033291722002859 (PMC10482711; doi:10.1017/S0033291722002859)
Supplement: Supplementary file 1 [file S0033291722002859sup001.docx]

**ONLINE SUPPLEMENTARY MATERIAL**

**Table of contents**

[**1.** **Supplementary methods** 3](#_Toc101207934)

[**Supplementary table 1.** Criteria for the classification and clinical prioritisation of relevant disproportionality signals 3](#_Toc101207935)

[**2.** **Supplementary results** 3](#_Toc101207936)

[**Supplementary table 2.** Mean prescribed daily dose for each antidepressant 3](#_Toc101207937)

[**Supplementary table 3.** Reporting odds ratios (ROR) and information components (IC) for withdrawal Antidepressants-related withdrawal syndrome by class of antidepressant and for each antidepressant using methadone as a positive control 4](#_Toc101207938)

[**Supplementary table 4.** Disproportionality intraclass analysis for tricyclic antidepressants 4](#_Toc101207939)

[**Supplementary table 5.** Disproportionality intraclass analysis for SSRIs 4](#_Toc101207940)

[**Supplementary table 6.** Disproportionality intraclass analysis for other antidepressants 5](#_Toc101207941)

**Supplementary table 7**. Comparison between serious and non-serious reactions in neonates without psychotropic medications other than antidepressants 5

[**Supplementary table 8.** Clinical priority evaluation and classification of relevant disproportionality signals 6](#_Toc101207942)

[**3.** **Supplementary references** 7](#_Toc101207943)

1. **Supplementary methods**

**Supplementary table 1.** Criteria for the classification and clinical prioritisation of relevant disproportionality signals

| **Clinical priority features for each drug** | **2 points** | **1 point** | **0 points** |
| --- | --- | --- | --- |
| Number of cases of withdrawal syndrome/Total number of reports of any AE | >10% | 5-10% | 0-4%: |
| Number of cases of withdrawal syndrome without confounders/number of all cases of withdrawal | ≥71% | 51-70% | <50% |
| Significant ROR and IC – consistent across different analyses  (in the main analysis, in the intraclass analysis and with methadone as a comparator) | ROR and IC significant in all three analyses | ROR and IC significant in two analyses | ROR and IC significant one analysis |
| Magnitude of the lower limit of the 95% CI of the ROR | .. | >10 | 0-10 |

AEs: adverse events*;* IC: information component; ROR: reporting odds ratio.

Confounders were defined as all drugs that can cause withdrawal syndrome, i.e., other psychotropic drugs, opioids, any other substance of abuse.

# **Supplementary results**

## **Supplementary table 2.** Mean prescribed daily dose for each antidepressant

| **Drug** | **n of cases with available dose** | **Daily dose (mg/day), median (Q1-Q3)** |
| --- | --- | --- |
| Amitriptyline | 10 | 50.0 (25.0-75.0) |
| Bupropion | 2 | 150.0 (150.0-150.0) |
| Citalopram | 20 | 20.0 (20.0-40.0) |
| Clomipramine | 24 | 75.0 (36.2-150.0) |
| Doxepin | 3 | 120.0 (110.0-160.0) |
| Duloxetine | 6 | 35.0 (22.5-55.0) |
| Escitalopram | 20 | 17.5 (10.0-20.0) |
| Fluoxetine | 25 | 30.0 (20.0-40.0) |
| Fluvoxamine | 4 | 62.5 (43.7-93.7) |
| Mianserin | 1 | 40.0 |
| Mirtazapine | 6 | 30.0 (18.7-41.2) |
| Paroxetine | 39 | 20.0 (20.0-38.7) |
| Sertraline | 27 | 50.0 (50.0-150.0) |
| Trazodone | 6 | 100.0 (62.5-175.0) |
| Venlafaxine | 35 | 150.0 (75.0-168.8) |

**Supplementary table 3.** Reporting odds ratios (ROR) and information components (IC) for antidepressant-related withdrawal syndrome by class of antidepressant and for each antidepressant using methadone as a positive control

| **Drug** | **n cases** | **n non-cases** | **ROR** | **Lower**  **95%CI** | **Upper 95%CI** | **IC** | **Lower 95%CI** | **Upper 95%CI** |
| --- | --- | --- | --- | --- | --- | --- | --- | --- |
| Antidepressants (All) | 379 | 2,817 | 0.07 | 0.06 | 0.10 | -0.42 | -0.59 | -0.30 |
| Tricyclic antidepressants | 69 | 238 | 0.16 | 0.11 | 0.23 | -0.90 | -1.30 | -0.61 |
| SSRIs | 238 | 2,060 | 0.06 | 0.05 | 0.08 | -0.62 | -0.83 | -0.46 |
| Others | 99 | 620 | 0.09 | 0.06 | 0.12 | -0.99 | -1.32 | -0.75 |
| **Tricyclic antidepressants** | | | | | | | | |
| Amitriptyline | 14 | 100 | 0.08 | 0.04 | 0.14 | -1.95 | -2.85 | -1.33 |
| Clomipramine | 47 | 117 | 0.22 | 0.14 | 0.34 | -0.82 | -1.30 | -0.47 |
| Doxepin | 5 | 10 | 0.27 | 0.09 | 0.83 | -0.85 | -2.41 | 0.13 |
| **SSRIs** | | | | | | | | |
| Citalopram | 33 | 307 | 0.06 | 0.04 | 0.09 | -1.78 | -2.36 | -1.37 |
| Escitalopram | 34 | 293 | 0.06 | 0.04 | 0.10 | -1.72 | -2.29 | -1.31 |
| Fluoxetine | 50 | 460 | 0.06 | 0.04 | 0.09 | -1.53 | -2.00 | -1.19 |
| Fluvoxamine | 7 | 33 | 0.11 | 0.05 | 0.27 | -1.67 | -2.97 | -0.82 |
| Paroxetine | 71 | 641 | 0.06 | 0.04 | 0.09 | -1.31 | -1.70 | -1.03 |
| Sertraline | 51 | 378 | 0.07 | 0.05 | 0.11 | -1.42 | -1.89 | -1.09 |
| **Other antidepressants** | | | | | | | | |
| Bupropion | 6 | 96 | 0.03 | 0.01 | 0.08 | -2.93 | -4.35 | -2.02 |
| Duloxetine | 8 | 84 | 0.05 | 0.02 | 0.11 | -2.46 | -3.67 | -1.66 |
| Mianserin | 4 | 5 | 0.44 | 0.11 | 1.68 | -0.47 | -2.24 | 0.61 |
| Mirtazapine | 10 | 62 | 0.09 | 0.04 | 0.18 | -1.90 | -2.98 | -1.17 |
| Trazodone | 6 | 25 | 0.13 | 0.05 | 0.33 | -1.55 | -2.97 | -0.64 |
| Venlafaxine | 67 | 338 | 0.11 | 0.08 | 0.16 | -1.10 | -1.51 | -0.81 |

CI: confidence/credibility interval; IC: information component; n cases: number of cases of withdrawal syndrome; n non-cases: number of other adverse reactions excluding withdrawal syndrome; ROR: reporting odds ratio; SSRI: selective serotonin reuptake inhibitors.

**Supplementary table 4.** Disproportionality intraclass analysis for TCAs

| **Drug** | **n cases** | **n non-cases** | **ROR** | **Lower**  **95%CI** | **Upper 95%CI** | **IC** | **Lower 95%CI** | **Upper 95%CI** |
| --- | --- | --- | --- | --- | --- | --- | --- | --- |
| Amitriptyline | 14 | 100 | 0.35 | 0.18 | 0.67 | -0.85 | -1.70 | -0.19 |
| Clomipramine | 47 | 117 | 2.21 | 1.25 | 3.89 | 0.35 | -0.09 | 0.73 |
| Doxepin | 5 | 10 | 1.78 | 0.59 | 5.40 | 0.51 | -1.02 | 1.50 |

CI: confidence/credibility interval; IC: information component; n cases: number of cases of withdrawal syndrome; n non-cases: number of other adverse reactions excluding withdrawal syndrome; ROR: reporting odds ratio; TCA: tricyclic antidepressants.

**Supplementary table 5.** Disproportionality intraclass analysis for SSRIs

| **Drug** | **n cases** | **n non-cases** | **ROR** | **Lower**  **95%CI** | **Upper 95%CI** | **IC** | **Lower 95%CI** | **Upper 95%CI** |
| --- | --- | --- | --- | --- | --- | --- | --- | --- |
| Citalopram | 33 | 307 | 0.92 | 0.62 | 1.35 | -0.09 | -0.63 | 0.36 |
| Escitalopram | 34 | 293 | 1.00 | 0.68 | 1.47 | 0.01 | -0.52 | 0.45 |
| Fluoxetine | 50 | 460 | 0.92 | 0.67 | 1.28 | -0.08 | -0.50 | 0.29 |
| Fluvoxamine | 7 | 33 | 1.86 | 0.81 | 4.25 | 0.69 | -0.57 | 1.57 |
| Paroxetine | 71 | 641 | 0.94 | 0.70 | 1.26 | -0.05 | -0.41 | 0.26 |
| Sertraline | 51 | 378 | 1.21 | 0.87 | 1.69 | 0.20 | -0.22 | 0.57 |

CI: confidence/credibility interval; IC: information component; n cases: number of cases of withdrawal syndrome; n non-cases: number of other adverse reactions excluding withdrawal syndrome; ROR: reporting odds ratio; SSRI: selective serotonin reuptake inhibitors.

**Supplementary table 6.** Disproportionality intraclass analysis for other antidepressants

| **Drug** | **n cases** | **n non-cases** | **ROR** | **Lower**  **95%CI** | **Upper 95%CI** | **IC** | **Lower 95%CI** | **Upper 95%CI** |
| --- | --- | --- | --- | --- | --- | --- | --- | --- |
| Bupropion | 6 | 96 | 0.35 | 0.15 | 0.83 | -1.16 | -2.54 | -0.23 |
| Duloxetine | 8 | 84 | 0.56 | 0.26 | 1.20 | -0.63 | -1.80 | 0.20 |
| Mianserin | 4 | 5 | 5.18 | 1.37 | 19.63 | 1.37 | -0.36 | 2.45 |
| Mirtazapine | 10 | 62 | 1.01 | 0.50 | 2.04 | 0.01 | -1.02 | 0.77 |
| Trazodone | 6 | 25 | 1.53 | 0.61 | 3.84 | 0.45 | -0.93 | 1.37 |
| Venlafaxine | 67 | 338 | 1.75 | 1.11 | 2.74 | 0.26 | -0.10 | 0.59 |

CI: confidence/credibility interval; IC: information component; n cases: number of cases of withdrawal syndrome; n non-cases: number of other adverse reactions excluding withdrawal syndrome; ROR: reporting odds ratio.

**Supplementary table 7.** Comparison between serious and non-serious reactions in neonates without psychotropic medications other than antidepressants

|  | **Serious reactions** | **Non-serious reactions** | **OR (95%CI)** | **p-value** |
| --- | --- | --- | --- | --- |
| n | 150 | 8 |  |  |
| Neonatal sex: Females (n) | 52 (34.7)^a^ | 2 (25.0)^b^ | 0.77  (0.07 to 5.60) | 1.00 |
| Neonatal age (days), median (Q1-Q3) | 1.0 (0.0-2.0) | 0.5 (0.0-1.3) | NA | 0.62 |
| Maternal DDD, median (Q1-Q3) | 1.0 (1.0-2.0)^c^ | 1.00 (1.0-2.3)^d^ | NA | 0.71 |
| Duration of the maternal antidepressant treatment (days), median (Q1-Q3) | 266.0  (220.5-277.5)^e^ | NA^f^ | NA | 0.29 |
| Duration of neonatal withdrawal syndrome (days), median (Q1-Q3) | 4.0  (2.0-10.0)^g^ | NA^h^ | NA | 0.86 |

CI: confidence interval; DDD: defined daily dose; NA: not applicable; OR: odds ratio; Q1: first quartile; Q3: third quartile.

^a^Missing data for 18 neonates

^b^Missing data for two neonates

^c^Data available for 87 neonates

^d^Data available for three neonates

^e^Data available for 35 neonates

^f^Data available for only one neonate

^g^Data available for 26 neonates

^h^Data available for two neonates

**Supplementary table 8.** Clinical priority evaluation and classification of relevant disproportionality signals

| **Drug** | n cases | **CRITERION 1** | | **CRITERION 2** | | **CRITERION 3** | | | | **CRITERION 4** | | **TOTAL SCORE** | **Priority level** |
| --- | --- | --- | --- | --- | --- | --- | --- | --- | --- | --- | --- | --- | --- |
|  |  | n cases /  total n AEs | SCORE | n cases without confounders/ n cases | SCORE | Significance across analyses | | | SCORE | Magnitude of ROR lower 95%CI | SCORE |  |  |
|  |  |  |  |  |  | Main | Intraclass | vs. methadone |  |  |  |  |  |
| Amitriptyline | 14 | 12.28% | 2 | 0.00% | 0 | 🗸 | x | x | 0 | 2.79 | 0 | **2** |  |
| Bupropion | 6 | 5.88% | 1 | 0.00% | 0 | x | x | x | 0 | 0.95 | 0 | **1** |  |
| Citalopram | 33 | 9.71% | 1 | 42.42% | 0 | 🗸 | x | x | 0 | 2.64 | 0 | **1** |  |
| Clomipramine | 47 | 28.66% | 2 | 23.40% | 0 | 🗸 | x | x | 0 | 10.22 | 1 | **3** |  |
| Doxepin | 5 | 33.33% | 2 | 0.00% | 0 | 🗸 | x | x | 0 | 5.93 | 0 | **2** |  |
| Duloxetine | 8 | 8.70% | 1 | 25.00% | 0 | 🗸 | x | x | 0 | 1.60 | 0 | **1** |  |
| Escitalopram | 34 | 10.40% | 2 | 32.35% | 0 | 🗸 | x | x | 0 | 2.86 | 0 | **2** |  |
| Fluoxetine | 50 | 9.80% | 1 | 36.00% | 0 | 🗸 | x | x | 0 | 2.88 | 0 | **1** |  |
| Fluvoxamine | 7 | 17.50% | 2 | 14.29% | 0 | 🗸 | x | x | 0 | 3.26 | 0 | **2** |  |
| Mianserin | 4 | 44.44% | 2 | 0.00% | 0 | 🗸 | x | x | 0 | 7.45 | 0 | **2** |  |
| Mirtazapine | 10 | 13.89% | 2 | 10.00% | 0 | 🗸 | x | x | 0 | 2.88 | 0 | **2** |  |
| Paroxetine | 71 | 9.97% | 1 | 54.93% | 1 | 🗸 | x | x | 0 | 3.11 | 0 | **2** |  |
| Sertraline | 51 | 11.89% | 2 | 25.49% | 0 | 🗸 | x | x | 0 | 3.58 | 0 | **2** |  |
| Trazodone | 6 | 19.35% | 2 | 16.67% | 0 | 🗸 | x | x | 0 | 3.42 | 0 | **2** |  |
| Venlafaxine | 67 | 16.54% | 2 | 37.31% | 0 | 🗸 | x | x | 0 | 5.50 | 0 | **2** |  |

AEs: adverse events; CI: confidence interval; n cases: number of cases of withdrawal syndrome; total n AEs: number of all adverse events; ROR: reporting odds ratio.

1. **Supplementary references**

WHO. MedDRA Hierarchy 2021. https://[www.meddra.org/how-to-use/basics/hierarchy](http://www.meddra.org/how-to-use/basics/hierarchy) (accessed February 2021)
